# The neutrophil migration induced by tumour necrosis factor alpha in mice is unaffected by glucocorticoids

**DOI:** 10.1080/09629359791929

**Published:** 1997-02

**Authors:** C. A. Cecilio, E. H. Costa, P. Ucelli, C. A. A. Chaves, M. C. Toffoli, C. A. Flores, F. Q. Cunha, S. H. Ferreira, W. M. S. C. Tamashiro

**Affiliations:** 1 Department of Microbiology and Immunology Institute of Biology UNICAMP Campinas SP 13081-970 Brazil; 2 Department of Pharmacology Faculty of Medicine UNICAMP Campinas SP 13081-970 Brazil; 3 Department of Pharmacology Faculty of Medicine UFCE Fortaleza CE 60001-970 Brazil; 4 Department of Pharmacology Faculty of Medicine of Ribeirão Preto USP Ribeirão Preto SP 14049 Brazil

## Abstract

Macrophages harvested from the peritoneal cavities of rats release a neutrophil chemotactic factor (MNCF) in response to stimulation with Gram-negative bacterial lipopolysaccharide (LPS). MNCF has been shown to be active in rats treated with dexamethasone, a glucocorticoid that usually inhibits the neutrophil migration induced in this species by interleukin (IL)-1, tumour necrosis factor alpha (TNFα), IL-8, C5a and leukotriene B_4_ (LTB_4_). Here we report that macrophages harvested from peritoneal cavities of mice, and stimulated *in vitro* with LPS, also release a factor that induces neutrophil migration in dexamethasone-treated animals. This chemotactic activity was neutralized by the incubation of the LPS-stimulated macrophage supernatants with a purified polyclonal IgG anti-mouse TNFα. In addition, significant amounts of TNF were detected in the supernatants. The neutrophil migration induced by intraperitoneal administration of recombinant murine TNFα was also unaffected by pretreatment of the mice with dexamethasone. Moreover, neutrophil migration induced by intraperitoneal injection of LPS was completely blocked by pretreatment of the mice with a monoclonal antibody against murine TNFα. In conclusion, our results support the hypothesis that, in contrast to the role of TNF in rats (where it indirectly induces neutrophil migration), in mice, it may be an important mediator in the recruitment of neutrophils to inflammatory sites.


Mediators of Inflammation, 6, 46–52 (1997)

### The neutrophil migration induced by tumour necrosis factor alpha in mice is unaffected by glucocorticoids

C. A. Cecilio,[1] E. H. Costa,[1] P. Ucelli,[1] C. A. A. Chaves,[1] M. C. Toffoli,[2] C. A. Flores,[3] F. Q. Cunha,[4] S. H. Ferreira[4] and W. M. S. C. Tamashiro[1,CA]

[1]Department of Microbiology and Immunology, Institute of Biology, UNICAMP, 13081-970, Campinas, SP, Brazil; [2]Department of Pharmacology, Faculty of Medicine, UNICAMP, 13081-970, Campinas, SP, Brazil; [3]Department of Pharmacology, Faculty of Medicine, UFCE, 60001-970, Fortaleza, CE, Brazil; [4]Department of Pharmacology, Faculty of Medicine of Ribeirão Preto, USP, 14049 Ribeirão Preto, SP, Brazil

[CA]Corresponding Author
Fax: (+55) 19 2393124

**MACROPHAGES harvested from the peritoneal cavities of rats release a neutrophil chemotactic factor (MNCF) in response to stimulation with Gram-negative bacterial lipopolysaccharide (LPS). MNCF has been shown to be active in rats treated with dexamethasone, a glucocorticoid that usually inhibits the neutrophil migration induced in this species by interleukin (IL)-1, tumour necrosis factor alpha (TNFα), IL-8, C5a and leukotriene $B_4$ ($LTB_4$). Here we report that macrophages harvested from peritoneal cavities of mice, and stimulated *in vitro* with LPS, also release a factor that induces neutrophil migration in dexamethasone-treated animals. This chemotactic activity was neutralized by the incubation of the LPS-stimulated macrophage supernatants with a purified polyclonal IgG anti-mouse TNFα. In addition, significant amounts of TNF were detected in the supernatants. The neutrophil migration induced by intraperitoneal administration of recombinant murine TNFα was also unaffected by pretreatment of the mice with dexamethasone. Moreover, neutrophil migration induced by intraperitoneal injection of LPS was completely blocked by pretreatment of the mice with a monoclonal antibody against murine TNFα. In conclusion, our results support the hypothesis that, in contrast to the role of TNF in rats (where it indirectly induces neutrophil migration), in mice, it may be an important mediator in the recruitment of neutrophils to inflammatory sites.**

**Key words:** Glucocorticoids, Neutrophil migration, Tumour necrosis factor alpha

### Introduction

Neutrophil migration is a key event in acute inflammatory reactions. This is a complex and multimediated event and remains not completely understood. Chemotactic mediators are generally derived either from plasma components or from multiple cellular sources,[1,2] and one of the most important cellular sources of neutrophil chemotactic factors is resident macrophages.[1,3] In fact, it has been shown that neutrophil migration into the peritoneal cavity, induced by carrageenin, endotoxin, latex beads or zymozan, is increased when the number of resident macrophages is augmented by pretreatment with thioglycollate, and reduced after depletion of the peritoneal macrophage population through previous washing of the cavity.[4]

The chemotactic mediators thought to be involved in neutrophil migration into inflamed tissues include the fifth complement component fragment, C5a,[5] leukotriene B4 ($LTB_4$),[6] and the pleitropic cytokines interleukin (IL)-1,[7] tumour necrosis factor alpha (TNFα),[7] and IL-8.[8] Some of these substances, such as C5a, may have a direct chemotactic effect on neutrophils;[5] others may act indirectly through mechanisms which depend upon the resident cells of the tissues, and the endothelium.[7,8] IL-1, TNF and $LTB_4$, for example, not only increase the expression of adhesion molecules but also stimulate the release of chemotactic factors by rat resident macrophages.[7] Furthermore, neutrophil migration into the peritoneal cavities of rats induced by these substances is also dependent on the number of resident macrophages.[7] Another potent leukocyte chemoattractant cytokine is IL-8, which has been shown to induce neutrophil migration by means of a mast cell dependent mechanism, since its activity cannot

© 1997 Rapid Science Publishers be observed in rat peritoneal cavities depleted of mast cells or in mice lacking this cellular population.[8] Furthermore, rat mast cells stimulated with IL-8 release a neutrophil chemotactic activity into the supernatant.[8]

It has been demonstrated that macrophages incubated *in vitro* with either LPS[3] or IL-1 and TNFα[7] release a neutrophil chemotactic factor named MNCF (macrophage-derived neutrophil chemotactic factor) into the supernatant. This factor has a mol.wt of 54 kDa, a PI < 4.0 and lectin-like activity.[9] The neutrophil migration induced by MNCF in rats is unaffected by the variation of the number of resident peritoneal cells, as well as by pretreatment of animals with glucocorticoids. Based upon these results, it was suggested that MNCF may be a mediator involved in the recruitment of neutrophil induced by various inflammatory stimuli, such as the cytokines IL-1 and TNFα in rats.

The fact that the glucocorticoids did not affect MNCF-induced neutrophil migration conflicts with the hypothesis that glucocorticoids block the expression of adhesion molecules in the neutrophil and endothelium.[10] It thus seems reasonable to speculate that certain stimuli, such as MNCF, can by-pass one or more critical steps which are sensitive to glucocorticoids, inducing leukocyte migration, whereas other stimuli, even though capable of by-passing some of these steps, cannot cause *in vivo* neutrophil migration in glucocorticoid-treated animals. Thus, it has been shown that in the hamster chick-pouch model of inflammation, dexamethasone failed to block the $LTB_4$-induced neutrophil adherence to and migration through endothelial cells.[11] In this model the neutrophils were halted in the lamina basal, between the endothelial cells and the pericyte, never reaching the intersticial space.

The present study shows that LPS-stimulated mouse macrophage monolayers release an MNCF-like mediator into culture supernatants since these supernatants induced neutrophil migration into the peritoneal cavities of animals pretreated with dexamethasone. Not only were significant amounts of TNFα deteted in the supernatants, but their *in vivo* neutrophil chemotactic activity was also neutralized by preincubation with a purified polyclonal IgG anti-mouse TNFα. Furthermore, the neutrophil recruitment induced by the intraperitoneal administration of recombinant murine TNFα was not affected by the treatment of the mice with dexamethasone. A monoclonal antibody against TNF was also shown to be able to block the LPS-induced neutrophil migration to the peritoneal cavities of mice completely.

### Materials and Methods

### Reagents

LPS isolated from *Escherichia coli* O111B4 was purchased from Sigma (St Louis, MO). Recombinant murine TNFα (rmTNFα) was donated by Dr M. A. Palladino (Genentech Inc., South San Francisco, CA). The rat monoclonal antibody against rmTNFα (XT22.11) was purified from mice ascitic peritoneal fluids by affinity chromatography on Sepharose-protein G (Sigma).

### Animals

Male 8- to 10-week-old C57BL/6 mice (Centro Multinstitucional de Bioterismo-CEMIB/UNICAMP, Campinas, SP, Brazil) were housed in a temperature-controlled room and received water and food *ad libitum* throughout the period of study.

### Preparation of anti-TNF sera

Polyclonal rabbit antisera against recombinant murine TNFα were produced in New Zealand rabbits, as described elsewhere.[12] Briefly, on day 0, 100 μg of rmTNFα were diluted in 500 μl of a phosphate buffered saline solution (PBS) and mixed with an equal volume of Freund's complete adjuvant, and the emulsion was injected intradermically into the rabbits. Boosting with intradermal injections of emulsions containing 50, 10, and 10 μg of rmTNF, in 50% Freund's incomplete adjuvant, occurred on days 15, 30 and 45, respectively. The rabbits were bled 10 days after the final injection. Specific antibodies against TNF present in the sera were detected by immunoblotting and capacity to block the effect of TNF on WEHI cells. In some experiments, antibodies obtained by chromatography of the sera in DEAE-Sepharose, according to the manufacturer's recommendations, were used as an IgG fraction.

### LPS stimulated murine macrophage supernatants

Mouse peritoneal cavities were stimulated by the injection of a 3% thioglycollate solution (DIFCO Laboratories; Detroit, MI) (3 ml/animal). Four days later, the peritoneal cells were harvested with RPMI 1640 (Flow Laboratories, McLean, VA) and seeded on plastic tissue culture dishes at a density of $2 \times 10^6$ cells/5 ml/plate. The cells were allowed to adhere for 1 h at 37°C, in an atmosphere of air with 5% $CO_2$. Adherent cells (95% macrophages) were washed three times with PBS (pH 7.2), and incubated for 30 min with 5 mg/ml of LPS in RPMI or with RPMI alone. The supernatants were discarded and the cells were extensively washed with PBS, followed by a final 2 h incubation at 37°C with 5 ml of RPMI medium per dish (without LPS or fetal calf serum). The cell-free incubation fluids were subsequently ultradiafiltered through a YM 10 membrane with a cut-off 10 Kd (Amicon Corp., Lexington, MA, USA). The final volumes (corresponding to 5% of the original volume) were filtered on 0.22 μm membranes (Millipore; Bedford, MA) and the samples were kept at −70°C for TNF detection and neutrophil migration assays.

### Detection of TNF in the supernatants

The TNF content in macrophage supernatants was measured using a highly TNF-sensitive cell line, WEHI 164 clone 13, as described elsewhere.[13] Briefly, WEHI cells were seeded on microplates (Costar 3596, Cambridge, MA) at a density of $4 \times 10^4$ cells/well in 50 μl of growth medium (RPMI 1640 medium containing 10% fetal calf serum (Gibco)). Fifty μl of diluted supernatants were added to the cells, and the experiments were conducted in triplicate. The plates were incubated for 20 h at 37°C in a 5% $CO_2$ incubator; 10 μl of a 3-4,5-dimethylthiazol-2-l-3,5-diphenylformazan solution (5 mg/ml PBS) (MTT, Sigma) were added to each well and the plates incubated for an additional 4 h. One hundred μl of isopropanol containing 0.04 N HCl were added to each well. Fifteen min later, the degree of cell lysis was determined spectrophotometrically (570 nm) using an enzyme-linked immunoassay analyser (Multiskan MCC/340 MKII, Flow Laboratories). TNF content in the assayed materials was calculated by comparison with standard curves which were determined with rmTNF (Genentech Inc.). The results correspond to the averages obtained in at least three independent experiments.

The presence of the TNF in the supernatants was confirmed by ELISA assays using the monoclonal antibody against TNF (XT 22.11) as the coating antibody (10 μg/ml) and the rabbit polyclonal antibodies (serum dilution 1:200) as the detecting antibody. The bound rabbit antibodies were revealed using an immunoperoxidase conjugate anti-rabbit IgG, and the chromogenic substrat OPD (Sigma). In our system, it was possible to detect amounts of TNF varying between 50 to 0.25 ng/ml, according to standard curves performed with mrTNFα.

### In vivo neutrophil migration

The method for testing *in vivo* neutrophil migration has been described elsewhere.[3] Briefly, LPS solutions (8–1000 ng/0.2 ml of saline solution/animal), rmTNFα (10–100 μg/0.2 ml/animal) and either LPS-stimulated or non-stimulated macrophage supernatants (equivalent to the products released by $2 \times 10^6$ adherent cells/0.2 ml/animal) were injected into the peritoneal cavities of mice pretreated subcutaneously (s.c.) with dexamethasone (0.5 mg/kg) 1 h previously. Controls received intraperitoneal (i.p.) injections of sterile saline solution. Six hours after these i.p. injections, the mice were sacrificed in a $CO_2$ chamber, and their peritoneal cavities washed with 3 ml of PBS containing 5 IU/ml of heparin; total and differential cell counts in the washing fluid were conducted as described elsewhere.[14] The results are expressed as means ± standard error of the mean (SEM) for the number of neutrophils per ml of peritoneal wash.

In some experiments, rmTNFα was incubated for 30 min at 37°C with polyclonal IgG anti-TNF (100 μg IgG/100 ng TNF) prior to the i.p. injections. The control groups for these experiments received i.p. injections of the same quantity of the antibody diluted in saline solution.

In other experiments, monoclonal antibody against TNF (XT22.11) was administrated intraperitoneally to mice (100 μg/cavity) 1 h before the i.p. injections of saline solution (0.2 ml/cavity) or LPS (200 ng/cavity), with control group receiving i.p. injections of saline solution prior to the inflammatory stimulus.

### Protein measurement

The protein contents of purified materials was determined according to the method of Hartree.[15]

### Statistical analysis

The data are presented as the means ± SEM, with statistical comparisons between the groups performed using the Student *t*-test for unpaired samples.

### Results

### Time course and dose dependence of LPS-induced neutrophil migration in mice

The intraperitoneal injection of increasing doses of LPS in naive mice induced a dose-dependent neutrophil migration with a bell-shaped curve

(Fig. 1A). The maximal migration was observed with a dose of 200 ng LPS/cavity, peaking between 8 and 12 h after the LPS administration, and returning to control levels after 48 h (Fig. 1B); the neutrophil migration induced by the dose of 1000 ng/cavity was less than that observed with the other doses tested.

### Effects of dexamethasone pretreatment of mice and pre-incubation of the LPS-stimulated macrophage supernatants with TNF specific antibodies on capacity to induce neutrophil migration

The supernatants from LPS-stimulated macrophages induced a significant neutrophil migration into the peritoneal cavities of naive mice, which was not significantly inhibited by the pretreatment of the animals with dexamethasone (Fig. 2A). The dexamethasone treatment, however, reduced the neutrophil migration induced by saline solution. On the other hand, the pre-incubation of these supernatants (from $2 \times 10^6$ adherent cells) with the IgG fraction of anti-rmTNFα serum (100 μg) eliminated their capacity to induce neutrophil migration into peritoneal cavities of dexamethasone-pretreated mice (Fig. 2B). The same quantity of IgG did not interfere with neutrophil migration in the control group.

### TNF concentration in the LPS-stimulated macrophage supernatants

The TNF concentration for five different samples of LPS-stimulated macrophage supernatants, derived from the culture of $2 \times 10^5$ adherent cells, varied from 1 to 14 ng as detected by activity and/or ELISA assays.

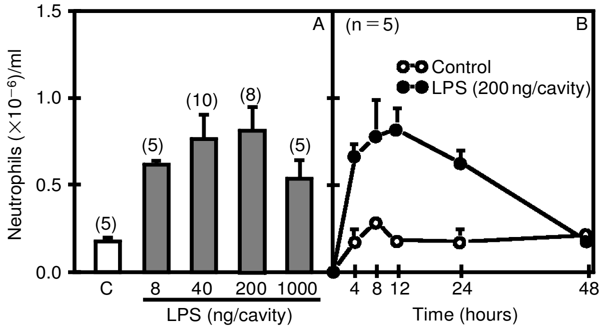

FIG. 1. Dose response and time course of neutrophil migration induced by LPS. (A) Bars represent neutrophil migration induced by the indicated doses of LPS into peritoneal cavities of mice. The control (C) represents neutrophil migration induced by saline solution. The neutrophil migration was analysed 6 h after the injection of the stimuli. The number of animals per experimental group is indicated at the top of the bars. (B) Time course of neutrophil migration induced by either LPS (200 ng/cavity) or saline solution (control group). The number of animals per group was five. In both panels, the results are reported as mean ± SEM.

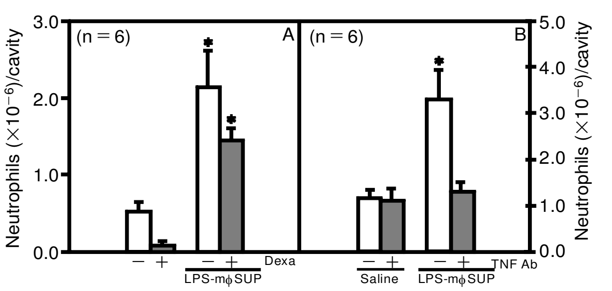

FIG. 2. Effects of dexamethasone pretreatment and preincubation of LPS-stimulated mouse macrophage supernatants with specific antibody to rmTNFα on capacity to induce neutrophil migration. (A) Bars represent neutrophil migration induced by saline solution or LPS-stimulated mouse macrophage supernatants (LPS-Mϕ SUP) into peritoneal cavities of mice, pretreated (+, hatched bars) or not (−, open bars) with dexamethasone (Dexa). (B) Bars represent neutrophil migration induced by LPS-Mϕ SUP, incubated (+) or not (−) with 100 μg of IgG anti-rmTNFα, into peritoneal cavities of dexamethasone-pretreated mice. Control groups received i.p. injections of saline solution or saline solution containing IgG anti-TNFα (100 μg/0.2 ml/animal). In both panels, the results are reported as mean ± SEM for six animals in the group. $^*P < 0.05$ (Student's t-test).

### Effect of dexamethasone pretreatment on neutrophil migration induced by recombinant murine TNFα in mice

The intraperitoneal administration of rmTNFα in naive mice induced a dose-dependent neutrophil migration which peaked 5–10 h after the injection. In dexamethasone-pretreated mice, rmTNFα also induced a dose-dependent neutrophil migration, which was determined 6 h later; the migration induced by a dose of 0.1 μg TNF/cavity in naive mice was similar to that observed for dexamethasone-pretreated animals (Fig. 3A). Fig. 3B shows that the neutrophil chemotactic activity of the rmTNFα was completely eliminated by pre-incubation with an anti-TNFα IgG.

### Effect of treatment with dexamethasone or TNF specific antibody on LPS-induced neutrophil migration in mice

The intraperitoneal injection of LPS in naive mice induced significant neutrophil migration which was suppressed by pretreatment with dexamethasone (Fig. 4A). This LPS-induced neutrophil migration was completely eliminated by pretreatment of the animals with a monoclonal antibody against TNFα (Fig. 4B).

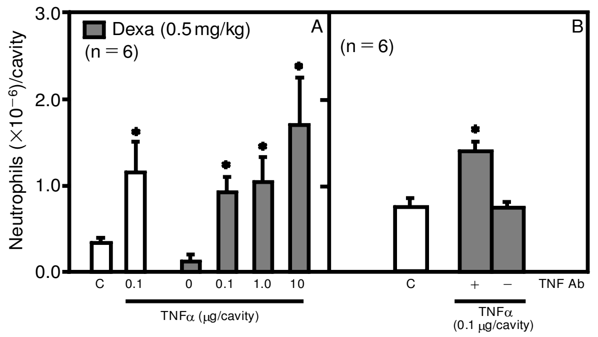

FIG. 3. Effect of dexamethasone pretreatment on the neutrophil migration induced by rmTNFα in mice. (**A**) Bars represent neutrophil migration induced i.p. injection of saline solution (SAL) or of the indicated amounts (in μg/animal) of rmTNFα into peritoneal cavities of dexamethasone-pretreated or naive mice (hatched and open bars, respectively). (**B**) Bars represent neutrophil migration induced by 0.1 μg rmTNFα, preincubated (+) or not (−) with 100 μg of IgG anti-TNFα, into peritoneal cavities of dexamethasone-pretreated mice. The control animals (SAL) received i.p. injections of saline solution containing IgG anti-TNFα (100 μg/0.2 ml/animal). In both panels, the results are reported as means ± SEM for six animals in the group. *P < 0.05 (Student's t-test).

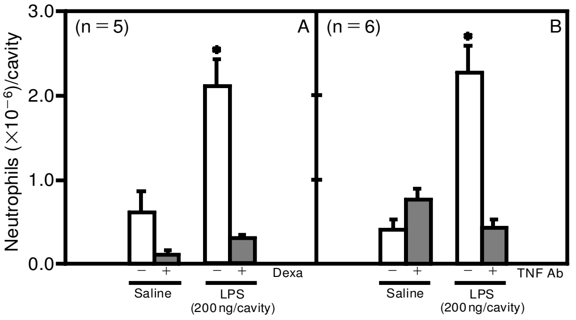

FIG. 4. Effects of pretreatment of mice with either dexamethasone or monoclonal antibody against murine TNFα on neutrophil migration induced by LPS. (**A**) Bars represent neutrophil migration induced by i.p. injection of saline solution (0.2 ml/cavity) or of LPS (200 ng/cavity) in dexamethasone-pretreated or non-treated mice (+, hatched and −, open bars, respectively). (**B**) Bars represent neutrophil migration induced by saline solution (0.2 ml/animal) or by LPS (200 ng/animal) into peritoneal cavities of mice either pretreated (+, hatched bars) or not (−, open bars) with monoclonal antibody against TNFα (100 μg/animal). In both panels, the results are reported as means ± SEM for five animals in the group. *P < 0.05 (Student's t-test).

### Discussion

The intraperitoneal injection of LPS in mice caused a significant, dose and time-dependent, neutrophil migration with a bell-shaped dose-response curve, as was found in other species.[16,17] This may be due to the liposolubility of the LPS and the fact that when it is injected in high concentrations into extravascular tissue, the amount that enters the circulatory system is sufficient to block neutrophil migration. It has, for example, been demonstrated that intravenous administration of LPS inhibits the neutrophil migration induced by various stimuli, including LPS itself.[16] It has recently been demonstrated that macrophages harvested from the peritoneal cavities of rats and stimulated *in vitro* with LPS release MNCF, a neutrophil chemotactic factor.[18] Rat macrophages stimulated *in vitro* with IL-1 and TNF also release MNCF.[7] The neutrophil migration induced by such MNCF is unaffected by the number of resident cells or by the treatment of the rats with dexamethasone.[19] The fact that this glucocorticoid did not affect the neutrophil migration induced by MNCF made the factor distinguishable from the other known neutrophil chemotactic mediators, since it has been demonstrated that the neutrophil migration induced by IL-1, TNF, IL-8, FMLP, C5a and $LTB_4$ in rats is inhibited by treatment with dexamethasone.[7,8] It is thus suggested that in rats MNCF may be a late mediator in the recruitment of neutrophil induced by various stimuli, including the cytokines IL-1 and TNF.

In the present study we demonstrated that macrophages harvested from peritoneal cavities of mice and stimulated with LPS for a brief period of time also released a neutrophil chemotactic factor into the supernatants, and the activity of this factor was not significantly inhibited by pre-treatment of animals with dexamethasone, at least at doses that block the neutrophil migration induced by LPS.

The incubation of the supernatant with an antibody against murine TNF blocked the neutrophil chemotactic activity, thus suggesting that the presence of the TNF was responsible for this activity. This fact also suggested that the neutrophil chemotactic activity of the supernatant was due to the presence of the TNF. The presence of TNF in the supernatant was then confirmed, and dose-dependent neutrophil migration in dexamethasone-treated mice was induced by the intraperitoneal administration of recombinant murine TNF. This neutrophil migration was not statistically different from that observed in untreated mice. The antibody that blocked the neutrophil chemotactic activity of the LPS-stimulated macrophage supernatant also eliminated it for rmTNF. The fact that the quantity of mrTNF necessary to induce neutrophil migration was greater than that found in the supernatant might be explained by the lack of glycosylation in the recombinant form, since it is well established that the level of glycosylation affects the specific activity of this cytokine. These results are in contrast to those obtained in rats, where the neutrophil migration induced by TNF is blocked by pre-treatment with dexamethasone; moreover, in this case TNF induces neutrophil migration via MNCF.[7]

In mice, the TNF generation upon LPS injection was suppressed by *in vivo* administration of a monoclonal antibody against TNF (XT22.11), which blocked the neutrophil migration induced by the endotoxin. It has recently been demonstrated that TNF is also involved in neutrophil migration into the lung induced by intratracheal challenge of presensitized mice with *Schistosoma mansoni* egg antigen.[20]

It is well documented that glucocorticoids inhibit the release of several cytokines, including TNF.[21–30] This fact could explain the inhibitory effect of dexamethasone on the neutrophil migration induced by LPS. The blockage of neutrophil-endothelium adhesion has been suggested as a mechanism by which the glucocorticoids inhibit the migration, although *in vitro* experimental evidences does not support this interpretation.[31] The *in vivo* monitoring of different stages of neutrophil migration showed that dexamethasone did not inhibit rolling or adhesion to the venular endothelium induced by $LTB_4$ and fMLP in hamsters.[32] Moreover, glucocorticoid treatment did not inhibit neutrophil penetration through the endothelial cell junctions. It was thus concluded that, in addition to the effect of corticoids on the release of chemotactic mediators, they blocked the final step of migration because the neutrophils remained between the endothelium and the basal membrane without reaching the perivascular tissue.[32] Haptotaxis seems to be an important mechanism in this final step, and the binding of the chemoattractant to a sub-endothelial or tissue matrix seems to be an important requirement for the process. In support of this hypothesis, *in vitro* evidence is provided to show that MNCF and IL-8 are able to induce migration through a haptotatic gradient, although they recognize different ligands.[33] Since dexamethasone blocks the neutrophil migration induced by IL-8 but does not affect the MNCF activity, it is postulated that glucocorticoids may be able to block the expression of the IL-8 ligand while not affecting that of MNCF.[33] Thus, TNF probably induces neutrophil migration in mice through a mechanism similar to that described for MNCF in rats, although the expression of the extravascular ligand to TNF in mice is not affected by glucocorticoid treatment. It is important to point out that, despite the fact that MNCF is not released in significant quantities by mouse macrophages, as demonstrated in the present study, when MNCF from rat macrophages was injected in dexamethasone pretreated mice, the neutrophil migration observed was similar to that observed in normal mice (data not shown).

In conclusion, the results reported here support the hypothesis that, in contrast to rats, in which TNF induces neutrophil migration through an indirect mechanism, in mice TNF may be a late mediator involved in the recruitment of neutrophil into an inflammatory site.

### References

1. Cybulsky MI, McComb DJ, Movat HZ. Protein synthesis dependent and independent mechanisms of neutrophil emigration. Different mechanisms of inflammation in rabbits induced by interleukin-1, tumor necrosis factor alpha or endotoxins versus leukocyte chemoattractants. *Am J Pathol* 1989; **135**: 227.
2. Kazmierosky JA, Gallin JI, Reynolds HY. Mechanism of the inflammatory response in primate lungs. Demonstration and partial characterization of an macrophage-derived chemotactic factor with preferential activity on polymorphonuclear leukocytes. *J Clin Invest* 1977; **59**: 273.
3. Cunha FQ, Ferreira SH. The release of a neutrophil chemotactic factor from peritoneal macrophages by endotoxin: inhibition by glucocorticoids. *Eur J Pharmacol* 1986; **139**: 65.
4. Souza GEP, Cunha FQ, Mello R, Ferreira SH. Neutrophil migration induced by inflammatory stimuli is reduced by macrophage depletion. *Agents and Actions* 1988; **24**: 377.
5. Yancey KB. Biological properties of human C5a: selected *in vitro* and *in vivo* studies. *Clin Exp Immunol* 1989; **71**: 207.
6. MacMillan RT, Foster SJ. Leukotriene B4 and inflammatory diseases. *Agents and Actions* 1988; **24**: 114.
7. Facioli LH, Souza GEP, Cunha FQ, Poole S, Ferreira SH. Recombinant interleukin-1 and tumor necrosis factor induce neutrophil migration 'in vivo' by indirect mechanisms. *Agents and Actions* 1990; **30**: 344.
8. Ribeiro RA, Flores CA, Cunha FQ, Ferreira SH. IL-8 causes in vivo neutrophil migration by a cell-dependent mechanism. *Immunology* 1991; **73**: 472.
9. Dias-Baruffi M, Cunha FQ, Ferreira SH, Roque-Barreira MC. Macrophage-released neutrophil chemotactic factor (MNCF) induces PMN-neutrophil migration through lectin-like activity. *Agents and Actions*, 1993; **38**: C54–C56.
10. Cronstein BN, Kimmel SC, Levein RI, Martiniuk F, Wiessman G. A mechanism for the antiinflammatory effects of corticosteroids: the glucocorticoid receptor regulates leukocyte adhesion molecule 1. *Proc Natl Acad Sci USA* 1992; **89**: 9991.
11. Katori M, Oda T, Nagai K. A site of action for dexamethasone on extravasation in the microcirculation. *Agents and Actions* 1992; **29**: 24.
12. Tamashiro WMSC, Taveres-Murta BM, Cunha FQ, Roque-Barreira MC, Nogueira RM, Ferreira SH. Neutrophil recruitment inhibitory factor: a possible candidate for a novel cytokine. *Mediators of Inflammation* 1992; **1**: 2.
13. Espevik T, Nissen-Mayer J. A highly sensitive cell line, WEHI 164 clone 13, for measuring citotoxic factor/tumor necrosis factor from human monocytes. *J Immunol Methods* 1986; **95**: 99.
14. Souza GEP, Ferreira SH. Blockade by antimacrophage serum of the migration of PMN neutrophils into inflamed peritoneal cavity. *Agents and Actions* 1985; **17**: 97.
15. Hartree EF. Determination of protein: a modification of the Lowry method that gives a linear photometric response. *Anal Biochem* 1972; **48**: 422.
16. Rocha N, Ferreira SH. Restoration by levamisole of endotoxin inhibited neutrophil migration, oedema and increased vascular permeability induced by carrageenin. *Eur J Pharmacol* 1986; **122**: 87.
17. Cunha FQ, Souza GEP, Souza CAM, Cerqueira BCS, Ferreira SH. In vivo blockage of neutrophil migration by LPS is mimicked by a factor released from LPS-stimulated macrophages. *Br J Clin Pharmacol* 1989; **36**: 485.
18. Cunha FQ, Souza GEP, Ferreira SH. Macrophages stimulated with lipopolysaccharide released a selective neutrophil chemotactic factor: an in vivo demonstration. *Brazilian J Med Biol Res* 1986; **19**: 775.
19. Souza GEP, Cunha FQ, Mello R, Ferreira SH. Neutrophil migration induced by inflammatory stimuli is reduced by macrophage depletion. *Agents and Actions* 1988; **4**: 377.
20. Lukacs NW, Strieter RM, Chensue SW, Widmer M, Kunkel SL. TNF-α mediates recruitment of neutrophils and eosinophils during airway inflammation. *J Immunol* 1995; **154**: 5411.
21. Arya SK, Wong-Staal F, Gallo RC. Dexamethasone-mediated inhibition of human T cell growth factor and gamma-interferon messenger RNA. *J Immunol* 1984; **133**: 273.
22. Culpepper J, Lee F. Regulation of IL-3 expression by glucocorticoids in cloned murine T lymphocytes. *J Immunol* 1985; **135**: 3191.

23. Culppeper J, Lee F. Glucocorticoid regulation of lymphokine production by murine T lymphocytes. *Lymphokines* 1987; **13**: 275.
24. Lew W, Oppenheim JJ, Matsushima K. Analysis of the suppression of IL-1β production in human peripheral blood mononuclear adherent cells by a glucocorticoid hormone. *Immunology* 1988; **140**: 1895.
25. Lee SW, Tsou AP, Chan H, Thomas J, Petrie K, Eugui EM, Allison AC. Glucocorticoids selectively inhibit the transcription of the interleukin 1β gene and decrease the stability of interleukin 1β mRNA. *Proc Natl Acad Sci USA* 1988; **85**: 1204.
26. Kern JA, Lamb RJ, Reed JC, Danielle RP, Owell PC. Dexamethasone inhibition of interleukin 1 beta production by human monocytes; post transcriptional mechanisms. *J Clin Invest* 1988; **81**: 237.
27. Nishida T, Nakai S, Kawakami T, Aihara K, Nishino N, Hirai Y. Dexamethasone regulation of the expression of cytokine mRNA induced by interleukin-1 in the astrocytoma cell line U373MG. *FEBS Lett* 1989; **243**: 25.
28. Mukaida N, Gusella GL, Kasahara T, Ko Y, Zachariae COC, Kawai T, Matsushima K. Molecular analysis of the inhibition of interleukin-8 production by dexamethasone in human fibrosarcoma cell line. *Immunology* 1992; **75**: 674.
29. Beutler B, Krochin N, Milsark IW, Luedke C, Cerami A. Control of cachectin (tumor necrosis factor) synthesis: mechanisms of endotoxin resistance. *Science (Wash DC)* 1986; **232**: 977.
30. Mukaida N, Zachariae COC, Gusella GL, Matsushima K. Dexamethasone inhibits the induction of monocyte chemotactic-activating factor production by IL-1 or tumor necrosis factor. *J Immunol* 1991; **146**: 1212.
31. Forsyth KD, Talbot V. Role of glucocorticoids in neutrophil and endothelial adhesion molecule expression and function. *Mediators of Inflammation* 1992; **1**: 101.
32. Oda T, Katori M. Inhibition site of dexamethasone on extravasation of polymorphonuclear leukocytes in the hamster cheek pouch microcirculation. *J Leukocyte Biol* 1992; **52**: 337.
33. Dias-Baruffi M, Roque-Barreira MC, Cunha FQ, Ferreira SH. Biological characterization of purified macrophage derived neutrophil chemotactic factor. *Mediators of Inflammation* 1995; **4**: 263.

ACKNOWLEDGEMENTS. This work was supported by grants from Fundação de Amparo à Pesquisa do Estado de São Paulo (FAPESP) and from Fundação de Apoio ao Ensino e Pesquisa da UNICAMP (FAEP) to W.M.S.C.T.

**Received 20 November 1996; accepted 26 November 1996**
